# Updated reference values for static lung volumes from a healthy population in Austria

**DOI:** 10.1186/s12931-024-02782-6

**Published:** 2024-04-03

**Authors:** Tobias Mraz, Shervin Asgari, Ahmad Karimi, Marie-Kathrin Breyer, Sylvia Hartl, Owat Sunanta, Alina Ofenheimer, Otto C. Burghuber, Angela Zacharasiewicz, Bernd Lamprecht, Caspar Schiffers, Emiel F. M. Wouters, Robab Breyer-Kohansal

**Affiliations:** 1 Department of Respiratory and Pulmonary Diseases, Vienna Healthcare Group, Clinic Penzing, Sanatoriumstrasse 2, Vienna, 1140 Austria; 2 Ludwig Boltzmann Institute for Lung Health, Vienna, Austria; 3 Faculty of Medicine, Sigmund Freud Private University, Vienna, Austria; 4 School of Nutrition and Translational Research in Metabolism, NUTRIM, Maastricht University Medical Center, Maastricht, the Netherlands; 5 Department of Pediatrics, Clinic Ottakring, Vienna, Austria; 6 Department of Pulmonology, Kepler University Hospital, Linz, Austria; 7 Medical Faculty, Johannes Kepler University, Linz, Austria; 8 Department of Respiratory and Pulmonary Diseases, Vienna Healthcare Group, Clinic Hietzing, Vienna, Austria

**Keywords:** Body plethysmography, Lung function, Lung volumes, Reference equations, General population

## Abstract

**Background:**

Reference values for lung volumes are necessary to identify and diagnose restrictive lung diseases and hyperinflation, but the values have to be validated in the relevant population. Our aim was to investigate the Global Lung Function Initiative (GLI) reference equations in a representative healthy Austrian population and create population-derived reference equations if poor fit was observed.

**Methods:**

We analysed spirometry and body plethysmography data from 5371 respiratory healthy subjects (6–80 years) from the Austrian LEAD Study. Fit with the GLI equations was examined using z-scores and distributions within the limits of normality. LEAD reference equations were then created using the LMS method and the generalized additive model of location shape and scale package according to GLI models.

**Results:**

Good fit, defined as mean z-scores between + 0.5 and -0.5,was not observed for the GLI static lung volume equations, with mean z-scores > 0.5 for residual volume (RV), RV/TLC (total lung capacity) and TLC in both sexes, and for expiratory reserve volume (ERV) and inspiratory capacity in females. Distribution within the limits of normality were shifted to the upper limit except for ERV. Population-derived reference equations from the LEAD cohort showed superior fit for lung volumes and provided reproducible results.

**Conclusion:**

GLI lung volume reference equations demonstrated a poor fit for our cohort, especially in females. Therefore a new set of Austrian reference equations for static lung volumes was developed, that can be applied to both children and adults (6–80 years of age).

**Supplementary Information:**

The online version contains supplementary material available at 10.1186/s12931-024-02782-6.

## Introduction

Respiratory disease conditions are largely based on measurement of lung physiology. A disease can be described as a set of characteristics by which they differ from the norm in such a way that they are biologically disadvantaged [1]. Reference values are used to help identify and diagnose individuals with abnormal values. Apart from measurement of forced maneuvers in spirometry, lung function can be described using lung volumes, determined by body plethysmography or gas dilution methods. Especially diagnosing restrictive lung disease only is possible by measuring the total lung capacity (TLC), thus requiring lung volumes [2].

The most commonly used reference values for lung volumes in adult populations are from the European Coal and Steel Community (ECSC), which were derived from data in 1983, and have limitations in terms of the inclusion of smokers and the lack of females [2, 3]. These are not applicable to children, and so separate reference values have to be used, the most common being based on work by Zapletal and colleagues published in the 1970s [3]. Values by Rosenthal et al. were also published more than 20 years ago [4]. Recognizing the need to update reference values for lung function testing, in 2012 the Global Lung Function Initiative (GLI) published multi-ethnic spirometry reference values that could be used across an age range of 3 to 95 years, with separate calculations for males and females [5]. Subsequently, the GLI published reference values for static lung volumes that are applicable to assessment either by gas dilution methods or plethysmography [6]. Whereas the GLI spirometry values are based on data from over 74,000 examinations and have been validated in a number of different populations [7], the static lung volume reference values are based on a more limited dataset of approximately 7,700 measurements [5, 6] and require further validation. We therefore aimed to investigate the fit of the GLI lung volume equations in a cohort of healthy never smokers in Austria. If resulting in a poor fit for the Austrian population, creation of population-derived reference equations was planned.

## Material and methods

### Population and study design

The LEAD (Lung, hEart, sociAl, boDy) Study (ClinicalTrials.gov; NCT01727518; http://clinicaltrials.gov) is an ongoing, longitudinal, observational, population-based cohort study that aims to provide a comprehensive database of risk factors for non-communicable diseases. The study has recruited a random sample (stratified by age, sex, and residential area) of males and females aged 6–80 years from Vienna and lower Austria that are representative of the general Austrian population, and who are being assessed every 4 years [8] since 2011. LEAD is being carried out according to the Declaration of Helsinki (2008) and has been approved by the Vienna local ethics committee (EK-11–117-0711). Written informed consent was given by all participants (or by parents or legal representatives for those aged under 18 years).

The current analyses focus on pre-bronchodilator data collected from the baseline visit. At each visit, all participants undergo spirometry and body plethysmography lung function testing by trained personnel at the LEAD study centre of the Ludwig Boltzmann Institute for Lung Health at the Clinic Penzing in Vienna, Austria. All measurements were conducted according to international recommendations (European Respiratory Society [ERS]/American Thoracic Society [ATS]) [9, 10], using BT-MasterScope Body 0478© (Jaeger, Germany) with the JLAB software. The body plethysmograph was calibrated daily using a 3 L syringe and a box pressure calibrator. Lung volume indices were expressed in body temperature pressure saturated conditions.

The lung function examination started with the subject sitting and breathing steadily, registering the pressure–flow diagrams, and producing at least three reproducible diagrams. Functional residual capacity (FRC) was then measured by closure of the shutter at the end of a normal expiration. At least two FRC loops were obtained, with the subject breathing against the shutter at resting ventilation. The subject then carried out a maximal expiration to measure expiratory reserve volume (ERV), with residual volume (RV) calculated by subtracting ERV from FRC, followed by a slow, maximal inspiration, from which inspiratory capacity (IC) was measured. Finally forced expiratory volume in 1 s (FEV_1_) and forced vital capacity (FVC) were assessed using forced spirometry, with three acceptable and reproducible loops obtained. Total lung capacity (TLC) was determined by adding RV to the best achieved vital capacity (VC), either from body plethysmography or spirometry. Strict regular quality control was in place for data collection and entry.

Age was registered in full days between the participants day of birth and the date of visit and is expressed in years with two decimals. Height was measured in centimeters without decimals. Weight was measured in kilograms with two decimals.

### Definition of healthy never smoking respiratory cohort

All current and ex-smokers were excluded from the analyses. Participants with respiratory symptoms (wheeze, cough, sputum, or dyspnoea) in the last 12-months were also excluded, obtained using an interview-based questionnaire. Further subjects with a doctor’s diagnosis of asthma, chronic obstructive pulmonary disease, chronic bronchitis, or emphysema were also excluded.

In order to avoid extreme outliers, patients with Z-scores ± 5 for height, weight or spirometric values were excluded from the analyses, and lung function reports of outliers were re-checked for errors and were evaluated for quality of the flow diagrams. Finally, we included only subjects with a complete set of pre- and post-bronchodilation spirometry and body plethysmography. As we believed this definition would describe pulmonary healthy subjects, no further exclusion criteria using spirometry or lung volumes were used.

To evaluate the cohort for single centre bias concerning pulmonary function testing, we included data from study participants, who underwent a second pulmonary function testing, using the same protocol, in the pulmonary function testing laboratory of the Clinic Penzing, Vienna. These were selected out of the initial study collective for bronchial challenge testing and do not necessarily correspond to the same subjects as in the healthy study cohort.

### Statistical analysis

Z-scores were calculated for the cohort using the available GLI reference equations for pre-bronchodilation spirometry and lung volumes [5, 6]. Spirometry was included to check for general comparability to the GLI cohorts. Fit was analysed using the mean Z-scores, the 95% confidence intervals and the percentage above the upper limit of normal (ULN) and below the lower limit of normal (LLN). A good fit was to be concluded if: 1) the mean Z-score was between + 0.5 and -0.5 2) the standard deviation (SD) was approximately 1; and 3) ≤ 5% of the observations were below the LLN and ≤ 5% were above the ULN [11].

Population-specific reference equations were created based on the same, healthy cohort using the LMS method, consistent with GLI [5], as described earlier by Cole et al. [12], and the generalised additive model of location, scale and shape (GAMLSS) package in R (Version 4.2.2, R Foundation, Vienna, Austria, http://www.r-project.org). Equations were generated separately for males and females, with height and age being the predictive variables. The LMS method allows modelling of the skewness (lamda), the median (mu) and the coefficient of variation (sigma). Fit of the equations was determined using Q-Q plots, worm plots and the distribution of Z-scores. The Kolmogorow-Smirnow test was used to test for normal distribution, indicated by a *p*-value > 0.05. Degrees of freedom were adapted to achieve the lowest Schwartz-Bayesian-Criterion while avoiding overly complex models.

## Results

**Fig. 1:**
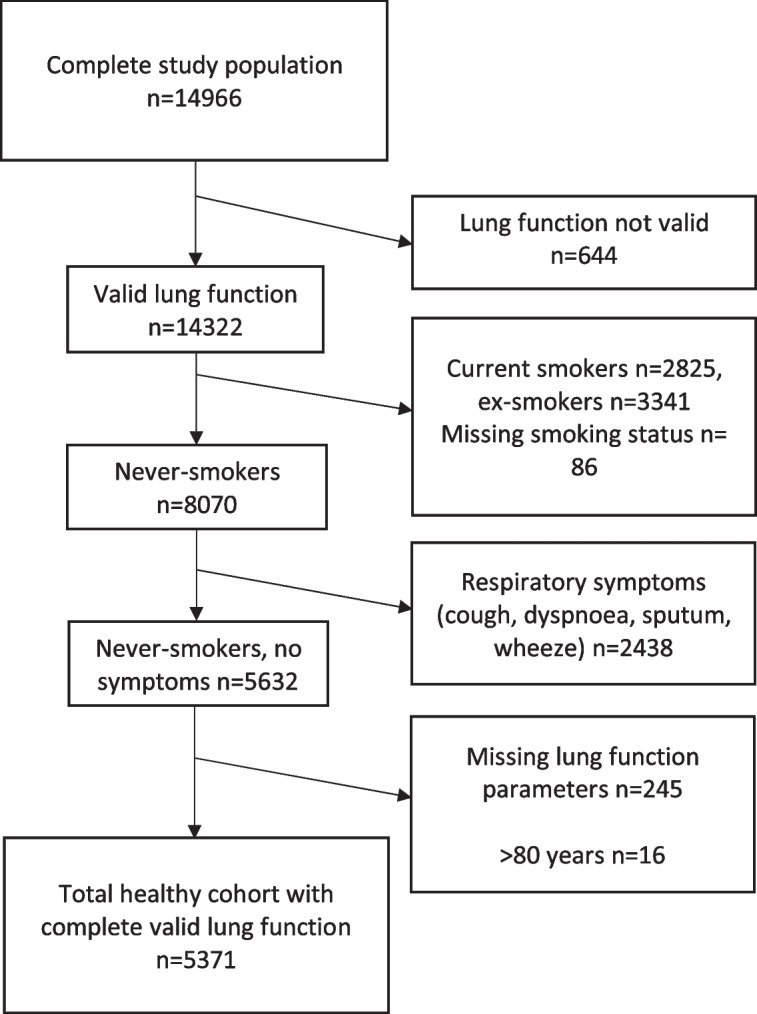
Flow chart for selection of a healthy, asymptomatic cohort

The analyses used data from 5371 subjects (Fig. 1), including 2397 males (43.9%) and 2974 females (56.1%), aged from 6 to 80 years. The baseline characteristics of this cohort are shown in Table 1 for males and Table 2 for females. The majority of included individuals were between 6 to 30 years. A decline of lung function could be observed for both sexes, but more pronounced for FEV1 and FVC than lung volumes. In contrast, RV, RV/TLC and FRC grow larger with increasing age.

**Table 1:** Baseline characteristics of the healthy cohort males

**Age Groups (years)**	6 to 10	≥ 10 to 20	≥ 20 to 30	≥ 30 to 40	≥ 40 to 50	≥ 50 to 60	≥ 60 to 70	≥ 70 to 80
*n* (total = 2397)	351	664	434	273	241	169	148	117
*mean (*± *SD)*								
** Height (cm)**	131.58 ± 8.64	163.44 ± 14.91	178.99 ± 6.98	178.68 ± 6.62	179.46 ± 7.31	179.01 ± 6.61	175.73 ± 6.93	173.48 ± 7.09
** Weight (kg)**	29.76 ± 7.61	56.17 ± 17.07	76.62 ± 12.50	81.54 ± 12.45	84.52 ± 13.65	85.36 ± 12.64	83.50 ± 11.55	82.28 ± 12.80
** FEV**_**1**_ ** (L)**	1.81 ± 0.34	3.35 ± 1.02	4.52 ± 0.62	4.30 ± 0.61	4.08 ± 0.57	3.85 ± 0.54	3.39 ± 0.52	3.05 ± 0.52
** FVC (L)**	2.07 ± 0.41	3.92 ± 1.18	5.44 ± 0.78	5.33 ± 0.80	5.25 ± 0.75	4.97 ± 0.69	4.44 ± 0.68	3.99 ± 0.68
** FEV**_**1**_ **/FVC (%)**	87.81 ± 6.13	85.63 ± 6.40	83.47 ± 7.00	80.99 ± 6.36	78.00 ± 5.55	77.69 ± 5.00	76.65 ± 6.04	76.59 ± 5.19
** TLC (L)**	3.03 ± 0.55	5.40 ± 1.57	7.43 ± 0.98	7.40 ± 1.08	7.55 ± 1.00	7.56 ± 1.03	7.35 ± 0.94	7.07 ± 1.03
** FRC (L)**	1.46 ± 0.29	2.64 ± 0.94	3.78 ± 0.73	3.64 ± 0.86	3.80 ± 0.83	3.86 ± 0.80	3.96 ± 0.74	3.82 ± 0.71
** RV (L)**	0.94 ± 0.26	1.45 ± 0.52	1.94 ± 0.46	2.01 ± 0.45	2.23 ± 0.41	2.49 ± 0.46	2.78 ± 0.46	2.92 ± 0.53
** RV/TLC (%)**	31.05 ± 6.39	26.97 ± 5.53	26.08 ± 4.86	27.03 ± 4.06	29.53 ± 3.49	32.80 ± 3.63	37.81 ± 4.43	41.47 ± 4.82
** IC (L)**	1.57 ± 0.37	2.76 ± 0.78	3.65 ± 0.61	3.76 ± 0.66	3.75 ± 0.64	3.70 ± 0.69	3.39 ± 0.61	3.25 ± 0.67
** ERV (L)**	0.51 ± 0.27	1.19 ± 0.61	1.84 ± 0.54	1.63 ± 0.61	1.57 ± 0.64	1.37 ± 0.56	1.18 ± 0.55	0.90 ± 0.44

*Abbreviations:* *ERV* expiratory reserve volume, *FEV1* forced expiratory volume in 1 s, *FVC* forced vital capacity, *FRCpleth* functional residual capacity measured by plethysmography, *IC* inspiratory capacity, *RV* residual volume, *TLC* total lung capacity

**Table 2:** Baseline characteristics of the healthy cohort females

**Age Groups (years)**	6 to 10	≥ 10 to 20	≥ 20 to 30	≥ 30 to 40	≥ 40 to 50	≥ 50 to 60	≥ 60 to 70	≥ 70 to 80
*n* (total = 2974)	564	794	493	285	291	218	190	139
*mean (*± *SD)*								
** Height (cm)**	131.18 ± 8.56	157.26 ± 10.45	165.7 ± 6.49	165.17 ± 6.58	165.77 ± 6.36	164.41 ± 6.30	161.71 ± 5.59	160.52 ± 5.71
** Weight (kg)**	29.54 ± 7.54	50.70 ± 13.70	61.49 ± 10.59	62.03 ± 10.39	67.63 ± 12.05	69.49 ± 13.13	69.55 ± 13.16	68.27 ± 11.39
** FEV**_**1**_ ** (L)**	1.72 ± 0.31	2.82 ± 0.59	3.31 ± 0.43	3.21 ± 0.44	3.01 ± 0.41	2.75 ± 0.39	2.50 ± 0.35	2.22 ± 0.35
** FVC (L)**	1.96 ± 0.37	3.22 ± 0.69	3.83 ± 0.53	3.87 ± 0.56	3.81 ± 0.53	3.51 ± 0.53	3.18 ± 0.64	2.86 ± 0.43
** FEV**_**1**_ **/FVC (%)**	88.54 ± 5.9	87.92 ± 5.82	86.62 ± 6.60	83.21 ± 6.83	79.41 ± 5.37	78.47 ± 5.03	78.79 ± 5.65	77.70 ± 5.06
** TLC (L)**	2.89 ± 0.51	4.54 ± 0.94	5.55 ± 0.71	5.71 ± 0.76	5.79 ± 0.77	5.74 ± 0.77	5.49 ± 0.65	5.36 ± 0.71
** FRC (L)**	1.38 ± 0.26	2.22 ± 0.58	2.92 ± 0.54	3.09 ± 0.60	3.12 ± 0.62	3.10 ± 0.61	3.00 ± 0.54	3.06 ± 0.56
** RV (L)**	0.92 ± 0.25	1.31 ± 0.41	1.68 ± 0.39	1.81 ± 0.4	1.94 ± 0.40	2.17 ± 0.36	2.25 ± 0,36	2.42 ± 0.43
** RV/TLC (%)**	31.9 ± 5.97	28.71 ± 6.17	30.08 ± 5.21	31.54 ± 5.00	33.34 ± 4.51	37.85 ± 3.84	40.97 ± 4.36	44.98 ± 4.61
** IC (L)**	1.51 ± 0.36	2.32 ± 0.54	2.63 ± 0.43	2.62 ± 0.45	2.67 ± 0.46	2.64 ± 0.45	2.49 ± 0.43	2.30 ± 0.41
** ERV (L)**	0.46 ± 0.23	0.91 ± 0.44	1.25 ± 0.39	1.29 ± 0.44	1.18 ± 0.45	0.93 ± 0.39	0.75 ± 0.39	0.65 ± 0.33

*Abbreviations:* *ERV* expiratory reserve volume, *FEV1* forced expiratory volume in 1 s, *FVC* forced vital capacity, *FRCpleth* functional residual capacity measured by plethysmography, *IC*, inspiratory capacity, *RV* residual volume, *TLC* total lung capacity

In the cohort, 31,2% of adults were overweight (body mass index [BMI] > 25 kg/m^2^) and 10,7% were obese (BMI > 30 kg/m^2^). In participants aged < 19 years 18,2% were overweight (BMI WHO Z-score > 1) and 9,6% were obese (BMI WHO Z-score > 2).

In a first step Z-scores were created using the GLI spirometry equations, to check for comparability to the Caucasian GLI cohorts. A good fit could be observed for all spirometry indices (Table 3). Females showed slightly lower numbers than the 5% expected under the LLN for FEV1 and FVC, especially at age > 65 years.

**Table 3:** GLI reference equations for spirometry in males and females of the LEAD cohort

	**Male (** ***n*** ** = 2397)**					**Female (** ***n*** ** = 2974)**				
	**Z–score**	**KS ** ***p*** **-value**	**95% CI**	**% > ULN**	**% < LLN**	**Z–score**	**KS ** ***p*** **-value**	**95% CI**	**% > ULN**	**% < LLN**
	**(mean ± SD)**					**(mean ± SD)**				
FEV_1_										
total	-0.03 ± 0.97	0.024	[-0.07, -0.01]	4.26	4.71	0.07 ± 0.99	< 0.001	[0.04, 0.11]	6.15	3.97
≤ 18 years	0.13 ± 1.00	0.001	[0.06, 0.19]	6.39	4.00	0.19 ± 1.04	< 0.001	[0.13, 0.25]	9.14	3.77
18–65 years	-0.19 ± 0.92	< 0.001	[-0.24, -0.14]	2.51	5.73	-0.09 ± 0.92	< 0.001	[-0.14, -0.04]	2.75	4.63
> 65 years	0.24 ± 0.92	0.016	[0.11, 0.36]	5.56	1.52	0.51 ± 0.90	< 0.001	[0.40, 0.63]	11.81	0.84
FVC										
total	-0.02 ± 0.96	0.04	[-0.06, 0.02]	3.55	4.71	0.10 ± 0.96	< 0.001	[0.07, 0.14]	5.08	3.73
≤ 18 years	0.12 ± 1.00	0.001	[0.06, 0.18]	5.19	3.68	0.23 ± 1.02	< 0.001	[0.18, 0.29]	7.70	3.93
18–65 years	-0.15 ± 0.92	< 0.001	[-0.20, -0.10]	1.96	5.88	-0.06 ± 0.90	< 0.001	[-0.11, -0.02]	2.48	4.09
> 65 years	0.20 ± 0.91	0.008	[0.07, 0.33]	6.06	2.02	0.43 ± 0.81	< 0.001	[0.32, 0.53]	7.59	0.42
FEV_1_/FVC										
total	-0.03 ± 0.98	0.002	[-0.07, 0.01]	4.92	4.63	-0.07 ± 0.97	< 0.001	[-0.10, -0.03]	5.52	4.84
≤ 18 years	0.01 ± 1.01	0.4	[-0.06, 0.07]	5.84	4.33	-0.11 ± 0.99	< 0.001	[-0.16, -0.05]	5.53	5.37
18–65 years	-0.07 ± 1.00	< 0.001	[-0.13, -0.02]	4.86	5.25	-0.05 ± 0.98	< 0.001	[-0.10, 0.00]	6.24	4.90
> 65 years	0.03 ± 0.75	0.056	[-0.08, 0.14]	1.01	2.02	0.03 ± 0.74	0.003	[-0.07, 0.12]	0.84	1.69

*Abbreviations:* *CI* confidence interval of mean Z-scores, *FEV*_*1*_ forced expiratory volume in 1 s, *FVC* forced vital capacity, *GLI* Global Lung Function Initiative, *KS* Kolmogorov–Smirnov test for distribution of mean Z-scores, *LLN* lower limit of normal, *ULN* upper limit of normal

### Existing reference equations for lung volume data

The fit of the GLI static lung volume equations were poor, as shown by the mean Z-scores in Table 4. Mean Z-scores for RV and RV/TLC using the GLI reference values were >  ± 0.5 for both males and females, with fit also poor for TLC, IC and ERV in females. Furthermore, there was a shift towards higher values for all indices except ERV, as indicated by a higher proportion of values above the ULN than below the LLN. A absent normal distribution was demonstrated for all indices by an *p* < 0.05 in the Kolmogorow-Smirnow test. An acceptable fit could be observed for FRC, IC and ERV in males, especially in the age group between 18–65 years.

**Table 4:** GLI reference equations for lung volumes in males and females

GLI^6^	Male (*n* = 2397)					Female (*n* = 2974)				
	**Z-score**	**KS ** ***p*** **-value**	**95% CI**	**% > ULN**	**% < LLN**	**Z-score**	**KS ** ***p*** **-value**	**95% CI**	**% > ULN**	**% < LLN**
	**(mean ± SD)**					**(mean ± SD)**				
TLC										
total	0.50 ± 0.96	< 0.001	[0.46, 0.54]	11.64	1.21	0.73 ± 0.93	< 0.001	[0.70, 0.77]	15.84	0.50
≤ 18 years	0.78 ± 099	< 0.001	[0.72, 0.84]	19.81	0.32	0.99 ± 0.97	< 0.001	[0.94, 1.04]	25.02	0.56
18–65 years	0.31 ± 0.90	< 0.001	[0.26, 0.36]	6.59	1.73	0.51 ± 0.85	< 0.001	[0.47, 0.56]	8.19	0.34
> 65 years	0.4 ± 0.91	< 0.001	[0.28, 0.53]	6.06	2.02	0.77 ± 0.85	< 0.001	[0.67, 0.88]	15.61	1.27
FRC										
total	0.36 ± 0.86	< 0.001	[0.32, 0.39]	6.09	1.75	0.45 ± 0.84	< 0.001	[0.42, 0.48]	6.83	1.01
≤ 18 years	0.33 ± 0.84	< 0.001	[0.28, 0.39]	5.63	1.95	0.37 ± 0.84	< 0.001	[0.32, 0.41]	5.37	1.20
18–65 years	0.39 ± 0.89	< 0.001	[0.34, 0.43]	6.67	1.80	0.5 ± 0.82	< 0.001	[0.46, 0.55]	7.45	0.94
> 65 years	0.28 ± 0.78	< 0.001	[0.17, 0.39]	4.55	0.51	0.52 ± 0.88	< 0.001	[0.41, 0.64]	10.55	0.42
RV										
total	0.68 ± 0.67	< 0.001	[0.65, 0.71]	7.63	0.04	0.91 ± 0.67	< 0.001	[0.89, 0.94]	12.88	0.17
≤ 18 years	0.60 ± 0.67	< 0.001	[0.56, 0.64]	4.98	0.11	0.84 ± 0.66	< 0.001	[0.80, 0.87]	9.94	0.40
18–65 years	0.75 ± 0.68	< 0.001	[0.71, 0.79]	9.80	0.00	1.01 ± 0.67	< 0.001	[1.05, 1.12]	16.11	0.00
> 65 years	0.61 ± 0.60	< 0.001	[0.52, 0.69]	6.06	0.00	0.72 ± 0.63	< 0.001	[0.44, 0.58]	8.02	0.00
RV/TLC										
total	0.81 ± 0.68	< 0.001	[0.78, 0.84]	11.39	0.00	0.96 ± 0.68	< 0.001	[0.94, 0.98]	14.46	0.13
≤ 18 years	0.80 ± 0.69	< 0.001	[0.76, 0.85]	11.15	0.00	0.89 ± 0.64	< 0.001	[0.86, 0.93]	9.54	0.24
18–65 years	0.84 ± 0.67	< 0.001	[0.81, 0.88]	12.31	0.00	1.09 ± 0.70	< 0.001	[1.05, 1.12]	20.60	0.07
> 65 years	0.62 ± 0.62	< 0.001	[0.53, 0.70]	6.57	0.00	0.51 ± 0.58	< 0.001	[0.44, 0.58]	1.69	0.00
IC										
total	0.30 ± 1.18	< 0.001	[0.25, 0.35]	12.60	3.09	0.62 ± 1.22	< 0.001	[0.58, 0.67]	19.33	1.14
≤ 18 years	0.82 ± 1.28	< 0.001	[0.74, 0.91]	24.68	2.06	1.27 ± 1.30	< 0.001	[1.19, 1.34]	38.17	1.04
18–65 years	-0.08 ± 0.95	< 0.001	[-0.13, -0.03]	4.24	3.84	0.10 ± 0.89	< 0.001	[0.06, 0.15]	4.50	1.41
> 65 years	0.30 ± 1.12	0.005	[0.14, 0.46]	10.10	3.03	0.51 ± 0.91	< 0.001	[0.39, 0.62]	13.50	0.00
ERV										
total	-0.34 ± 1.12	< 0.001	[-0.38, -0.29]	2.09	12.10	-0.56 ± 1.38	< 0.001	[-0.61, -0.51]	2.66	17.75
≤ 18 years	-0.62 ± 1.34	< 0.001	[-0.70, -0.53]	3.35	21.00	-1.03 ± 1.69	< 0.001	[-1.13, -0.94]	4.49	31.76
18–65 years	-0.14 ± 0.91	< 0.001	[-0.19, -0.09]	1.49	6.51	-0.24 ± 0.97	< 0.001	[-0.29, -0.19]	1.28	7.92
> 65 years	-0.32 ± 0.88	< 0.001	[-0.45, -0.20]	0.00	6.57	-0.17 ± 0.92	0.079	[-0.29, -0.06]	1.69	5.91

*Abbreviations:* *CI* confidence interval of mean Z-scores, *ERV* expiratory reserve volume, *FRC* functional residual capacity, *GLI* Global Lung Function Initiative, *IC*, inspiratory capacity, *KS* Kolmogorov–Smirnov test for distribution of mean Z-scores, *LLN* lower limit of normal, *RV* residual volume, *TLC* total lung capacity, *ULN* upper limit of normal

### Creation of population-specific reference equations

Given the unsatisfactorily fit of the lung volume data when using the GLI reference equations, new equations were created using the LMS method (Table 5, Supplementary Figures. 1 and 2). Consistent with the approach used by GLI, subjects with calculated Z-scores >  ± 5 were excluded before recalculating the equations, to avoid influence by extreme outliers. Look-up tables containing the varying coefficients were created and are available in the online supplement. All equations showed a good fit, with mean Z-scores of 0 and SDs of 1 (Table 6). Furthermore, all distributions were even with approximately 5% of subjects above and below ULN and LLN, respectively. All indices were normally distributed in the Kolmogorow-Smirnow test.

**Table 5:** Static lung volume equations for males and females (LEAD 2021)

	**Sex**	**Median (M)**	**Variability around the median (S)**	**Skewness (L)**
**TLC**	male	exp( -0.9506 + 0.01358 * height + 0.1428 * ln(age) + mu-spline)	exp( -2.122—0.036 * ln(age) + sigma-spline)	1
	female	exp( -9.509 + 2.121* ln(height) + 0.109* ln(age) + mu-spline)	exp( -2.0256—0.0712 * ln(age) + sigma-spline)	0.464
**FRC**	male	exp(-1.9725 + 0.0153 * height + 0.1537 * ln(age) + mu-spline)	exp(-2.0920 + 0.0981 * ln(age) + sigma-spline)	0.7562
	female	exp(-2.02250 + 0.01494* height + 0.17902 * ln(age) + mu-spline)	exp(-1.98221 + 0.04072 * ln(age) + sigma-spline)	1
**RV**	male	exp(-2.2895 + 0.0122* height + 0.2502* ln(age) + mu-spline)	exp(-0.6544—0.2846* ln(age) + sigma-spline)	1.4778—0.3313 * ln(age)
	female	exp( -2.3026 + 0.0123* height + 0.2516* ln(age) + mu-spline)	exp( -0.707—0.265 * ln(age) + sigma-spline)	1.685—0.369 * ln(age)
**RV/TLC**	male	exp(3.2352—0.0010 * height + 0.09698 * ln(age) + mu-spline)	exp(-0.8449—0.3138 * ln(age) + sigma-spline)	3.644—1.032 * ln(age)
	female	exp (3.258—0.0012 * (height) + 0.131 * ln(age) + mu-spline)	exp(-0.9245—0.292* ln(age) + sigma-spline)	2.53—0.5187* ln(age)
**IC**	male	exp(-9.504 + 2.001* ln(height) + 0.111* ln(age) + mu-spline)	exp( -1.68196—0.03498* ln(age) + sigma-spline)	0.6339
	female	exp(-8.5626 + 1.8228 * ln(height) + 0.0560 * ln(age) + mu-spline)	exp( -1.4862—0.09097 * ln(age) + sigma-spline)	0.7076
**ERV**	male	exp( -3.40565 + 0.01892 * height + 0.11655 * ln(age) + mu-spline)	exp( 0.3466—0.3694 * ln(age) + sigma-spline)	2.4951–0.4487 * ln(age)
	female	exp( -3.26931 + 0.01919 * height + 0.03159 * ln(age) + mu-spline)	exp(-0.6865—0.0707 * ln(age) + sigma-spline)	0.7913

*Abbreviations: ERV *expiratory reserve volume,* FEV*_*1 *_forced expiratory volume in 1 s,* FVC *forced vital capacity,* FRC *functional residual capacity,* IC *inspiratory capacity,* RV *residual volume,* TLC *total lung capacity, Mu-Spline and sigma-Spline correspond to the age-varying coefficients, available as a look-up table in the online supplement

**Table 6:** LEAD reference equations for lung volumes in males and females

LEAD	Male (*n* = 2397)					Female (*n* = 2974)				
	**Z-score**	**KS ** ***p*** **-value**	**95% CI**	**% > ULN**	**% < LLN**	**Z-score**	**KS ** ***p*** **-value**	**95% CI**	**% > ULN**	**% < LLN**
	**(mean ± SD)**					**(mean ± SD)**				
TLC										
total	0.00 ± 1.00	0.5	[-0.04, 0.04]	4.96	5.01	0.00 ± 1.00	0.4	[-0.04, 0.04]	5.08	5.21
≤ 18 years	0.00 ± 1.00	0.6	[-0.06, 0.07]	5.30	4.87	0.00 ± 1.01	0.4	[-0.05, 0.06]	5.61	4.81
18–65 years	0.00 ± 0.99	> 0.9	[-0.06, 0.05]	4.63	4.86	0.00 ± 0.98	0.14	[-0.05, 0.05]	4.30	5.44
> 65 years	0.01 ± 1.10	> 0.9	[-0.14, 0.17]	5.56	6.57	0.00 ± 1.06	0.7	[-0.13, 0.14]	7.17	5.91
FRC										
total	0.00 ± 1.00	0.7	[-0.04, 0.04]	5.13	4.96	0.00 ± 1.00	0.2	[-0.04, 0.04]	5.28	4.51
≤ 18 years	0.00 ± 1.00	0.5	[-0.06, 0.07]	5.09	5.09	0.00 ± 1.01	0.3	[-0.06, 0.05]	6.01	3.53
18–65 years	-0.01 ± 1.00	0.8	[-0.07, 0.04]	5.10	5.10	0.00 ± 0.99	0.9	[-0.05, 0.05]	4.70	5.23
> 65 years	0.06 ± 0.99	0.6	[-0.08, 0.19]	5.56	3.54	0.01 ± 1.03	0.6	[-0.12, 0.14]	5.06	5.06
RV										
total	0.00 ± 1.00	0.5	[-0.04, 0.04]	4.92	4.30	0.00 ± 1.00	0.081	[-0.04, 0.04]	5.99	4.10
≤ 18 years	-0.01 ± 0.99	0.5	[-0.08, 0.05]	4.22	4.22	0.00 ± 1.00	0.3	[-0.06, 0.05]	5.77	3.69
18–65 years	0.00 ± 1.00	0.9	[-0.05, 0.06]	5.18	4.24	0.00 ± 0.99	0.2	[-0.05, 0.05]	5.97	4.36
> 65 years	0.02 ± 1.02	0.7	[-0.13, 0.16]	6.57	5.05	0.01 ± 1.06	0.5	[-0.13, 0.14]	7.17	4.64
RV/TLC										
total	0.00 ± 1.00	0.2	[-0.04, 0.04]	4.63	4.55	0.00 ± 1.00	0.7	[-0.04, 0.04]	5.55	4.47
≤ 18 years	-0.01 ± 0.99	0.4	[-0.08, 0.05]	4.11	4.87	-0.01 ± 1.00	> 0.9	[-0.06, 0.05]	4.89	4.41
18–65 years	0.01 ± 1.00	> 0.9	[-0.05, 0.06]	5.10	4.39	0.00 ± 1.00	0.3	[-0.05, 0.06]	6.04	4.43
> 65 years	0.00 ± 1.00	0.5	[-0.13, 0.14]	4.04	4.04	0.00 ± 1.05	0.8	[-0.14, 0.13]	5.91	5.06
IC										
total	0.00 ± 1.00	0.15	[-0.04, 0.04]	5.26	5.05	0.00 ± 1.00	0.8	[-0.04, 0.04]	5.18	4.91
≤ 18 years	0.00 ± 1.00	0.5	[-0–07, 0.06]	5.09	4.87	0.00 ± 1.00	0.9	[-0.05, 0.06]	5.05	5.21
18–65 years	0.01 ± 1.00	0.6	[-0.04, 0.07]	5.65	4.94	0.00 ± 0.99	0.7	[-0.05, 0.05]	5.30	4.43
> 65 years	-0.06 ± 1.00	0.4	[-0.20, 0.08]	3.54	6.57	-0.02 ± 1.03	0.6	[-0.15, 0.11]	5.06	6.33
ERV										
total	0.00 ± 0.99	0.9	[-0.04, 0.04]	5.42	4.88	0.00 ± 1.00	0.3	[-0.04, 0.04]	4.77	4.71
≤ 18 years	0.02 ± 0.97	0.5	[-0.04, 0.08]	5.19	3.57	0.00 ± 0.99	0.3	[-0.05, 0.06]	5.05	4.41
18–65 years	-0.02 ± 1.01	> 0.9	[-0.07, 0.04]	5.41	5.88	0.00 ± 1.00	0.3	[-0.05, 0.05]	4.50	4.83
> 65 years	0.04 ± 1.01	> 0.9	[-0.10, 0.18]	6.57	4.55	-0.02 ± 1.02	0.4	[-0.15, 0.11]	5.06	5.49

*Abbreviations:* *CI* confidence interval of mean Z-scores, *ERV *expiratory reserve volume, *FRC* functional residual capacity, *IC* inspiratory capacity, *KS* Kolmogorov–Smirnov test for distribution of mean Z-scores, *LLN* lower limit of normal, *RV* residual volume, *TLC* total lung capacity, *ULN* upper limit of normal

### Intraindividual variability

As this was a single centre study, a measurement bias by operator or equipment couldn’t be excluded. However, a subgroup of the LEAD cohort underwent an additional pulmonary function testing at a different site: participants with history of atopy, allergy, eosinophilia or positive skin prick test were selected for a bronchial challenge testing, which was carried out at the pulmonary function lab of the Clinic Penzing. The protocol and equipment were the same type as in the study centre, being a BT-MasterScope Body 0478 (Jaeger, Germany). Normal spirometry and plethysmography were carried out, tough only TLC, RV and ERV were available in the database. During Phase 1765 individuals underwent the additional testing, after excluding all with missing or invalid data, 706 participants remained. As the mean interval between the measurements was 40 months, a manual quality check was carried out, to exclude children and adolescents with large differences between the dates due to natural growth, contributing to the high number of exclusions. In the end, data of 602 participants were analysed. As the mean intraindividual difference was < 100 ml for all included parameters (FEV1, FVC, ERV, RV, TLC), a single centre bias of measurements seemed unlikely. (Table 7).

**Table 7:** Mean measured values and intrasubject variations between two visits

	1st Visit (LEAD study center)	2nd Visit (Clinic Penzing)	Mean intraindividual difference
FEV_1_ (L)	3.70 ± 0.89	3.66 ± 0.87	-0.03 (± 0.22)
**FVC (L)**	4.53 ± 1.14	4.43 ± 1.13	-0.10 (± 0.32)
**RV (L)**	1.92 ± 0.59	1.93 ± 0.6	0.00 (± 0.33)
**TLC (L)**	6.52 ± 1.47	6.54 ± 1.41	0.02 (± 0.36)
**ERV (L)**	1.40 ± 0.61	1.42 ± 0.61	0.03 (± 0.35)

Data are mean (± SD)

Abbreviations: *ERV* expiratory reserve volume, *FEV*_*1*_ forced expiratory volume in 1 s, *FVC* forced vital capacity, *RV* residual volume, *TLC* total lung capacity

## Discussion

These analyses use cross-sectional data obtained from a broad, representative healthy population sample from Austria to investigate the fit of the GLI lung volumes reference equations. As the GLI equations failed to demonstrate a good fit with our population-based data in normal subjects, a new set of sex-specific reference values was created for lung volumes.

Reference values are indispensable when interpreting lung volumes in clinical practice, using the LLN with TLC and ULN with RV for defining restrictive impairment and hyperinflation respectively [13]. Until recently, assessments in Austria and Europe relied mostly on the ECSC reference equations for adults, despite several studies having demonstrated inconsistencies between these reference equations, so the update by GLI was highly anticipated [14–16].

When using the GLI spirometry equations in our population a good fit was observed. We therefore considered our cohort comparable to the Caucasian cohorts used by GLI to create equations for spirometry and lung volumes. While small differences exist especially for females, we consider the equations sufficient for the detection of obstructive anomalies in our cohort [17]. This is consistent with previous analyses reporting a good fit with the GLI spirometry equations for other European cohorts [7, 18]. While some authors still report significant differences [19], the GLI equations, at least for Caucasian populations, offer consistent cut-offs and improved comparability between cohorts. The large amount of collated data, smoothing out small differences between populations, seems one of the main advantages. Additionally, even ethnic-specific equations created by GLI are available for spirometry. But the accuracy of these compared to globally merged equations was questioned lately [20].

However, GLI lung volume reference values did not fit well within our cohort. Large differences were observed, with mean Z-scores > 0,5 for TLC, RV and RV/TLC. Also, the percentage under the LLN and over the ULN was lower and higher respectively than expected. The difference was even more pronounced in females including significant differences for IC and ERV. These deviations could lead to an under-detection of restrictive disorders and overdiagnosis of hyperinflation in the Austrian population.

So far there is few data about the performance of the new GLI equations in European cohorts. The number of observations for lung volumes was much lower than for spirometry, and no equations are available for different ethnic backgrounds than Caucasian. A recent study from Belgium found similar results, with the GLI equations underestimating especially the values for RV [21]. Furthermore, the percentage under the LLN was lower than the expected 5% for TLC. A study in Algerian adults also reported, despite good fitting GLI spirometry values, similar results for RV, RV/TLC and TLC [22].

One potential explanation for the poor fit of the GLI lung volume equations is that our data were collected recently (starting 2011). Longitudinal studies have shown that populations are getting taller and healthier [23], with average population lung function increasing [24–27], potentially influenced by socioeconomic factors, or reduced occupational or environmental exposure [25, 28]. While in literature the impact of these developments in lung function is still discussed, the large size of our cohort might especially contribute to visible differences [29].

There were less obese and overweight individuals in our cohort compared to GLI. As the significance of weight as predictor of static lung volumes is not yet conclusively understood [6], we used weight as an predictive variable in an early version of the equations. This only minimally altered the coefficients, and so wasn’t used further (data not shown). While weight seems to have only a small impact on overall lung volume reference equations, the effect of body composition could be more important and may explain some of the differences between cohorts.

Future analyses could investigate and include the effect of body compartments on lung volumes.

Other factors contributing to the need to revisit equations could be changes in methods and equipment. Various studies in patients with obstructive lung diseases have demonstrated significant differences between lung volumes measured by gas dilution methods versus plethysmography, although the situation in healthy individuals is less clear [30]. Indeed, GLI found statistically significant differences between these two methods in their cohort, but regarded the differences as not clinically relevant, although the majority of their data were derived from plethysmography [6]. In addition, use of different body plethysmography devices and software could potentially impact the results. For example, in GLI devices manufactured by JAEGER (which we used in our study) measured somewhat higher values than those from other manufacturers, especially for RV [6]. Recently, authors from COSYCONET demonstrated differences in FRC up to 0.67 L between two manufacturers [31].

So, while the simplicity of one equation spanning different techniques, equipment, and populations is one argument for the use of the GLI equations, this might not appropriately represent all different populations and methods. It is to be expected that reference values derived directly from the specific examined population would fit that population better than standardised equations – although it is important that for such population-based equations to be useful, the examined population has to be representative of the broad population, as has been shown to be the case with the LEAD cohort [8] Still, adding more data to the GLI equations, may in the future improve the generalizability and render population based equations obsolete.

In this study the population derived reference equations from LEAD demonstrated a superior fit for all lung volume indices compared to the GLI equations. Lung volumes in our cohort were influenced by sex, age and height. Some studies have included weight as predictive variable for lung function [15, 16, 32], but as with GLI we found only a small influence of weight [6], and our equations therefore do not need to include this parameter. Importantly, we included obese individuals in our analyses, since reference values should be generalisable to the intended population [17]. Our newly derived equations might be usable in other European countries with similar population characteristics and equipment. This will have to be analysed in future studies.

### Strengths and limitations

Our analyses were conducted according to the ERS/ATS workshop report requirements [2]. While these published already over 20 years ago, they are still the most recent criteria available. We used strict selection criteria for our healthy cohort, only including never smokers, and excluding those reporting any respiratory symptoms. In addition, the population was distributed over all age groups, although with an overrepresentation of children, adolescents and of females, potentially due to the exclusion of those with a smoking history. We used standardised methods for the measurement of lung volumes, with strict quality control [8], and to create the reference equations we used the same statistical models as GLI. In particular, the LMS model allows the equations to cover the entire age range, avoiding discrepancies when entering the adult age [5].

The main limitation of our analyses is the single centre aspect of our lung function testing. The comparison of measurements done in another site showed only very small, not clinically relevant differences. Still a systemic bias can’t be ruled out, as only the device and software of one manufacturer was used. This also limits generalisability to other equipment and software. Furthermore, our cohort included no individuals aged < 6 years and > 80 years, so we recommend the use of our equations only between the ages of 6 and 80 years. Ethnicity wasn’t documented, as participants of the LEAD study, corresponding to the Austrian population, were predominantly of European ancestry. The Austrian population is known to consist just a very minor part of subjects different than Caucasian ancestry, so ethnicity wasn’t considered in the initial study design. Therefore the reference values are only applicable to similar Caucasian populations. We used strict exclusion criteria, but still subjects with physiologically abnormal lung function measurements or undiagnosed respiratory disease could have been present in the analysed cohort.

## Conclusion

In our cohort the GLI lung volume reference equations demonstrated a poor fit for RV, RV/TLC and TLC, especially in females. We therefore developed a new set of Austrian reference equations for static lung volumes that, unlike most reference values, can be applied to both children and adults, from the ages of 6 to 80 years.

### Supplementary Information

**Additional file 1.** Mean predicted lung volumes males

**Additional file 2.** Mean predicted lung volumes females

**Additional file 3.** LEAD Lookup tables lung volumes submitted

## Data Availability

The reference equations generated and analysed during the current study are available in Table 5. Look-up tables are provided in the online supplement.
