# Supplementary figures and images for: Updated reference values for static lung volumes from a healthy population in Austria
Source: Respir Res. 2024 Apr 3;25:155. doi: 10.1186/s12931-024-02782-6 (PMC10988832; doi:10.1186/s12931-024-02782-6)

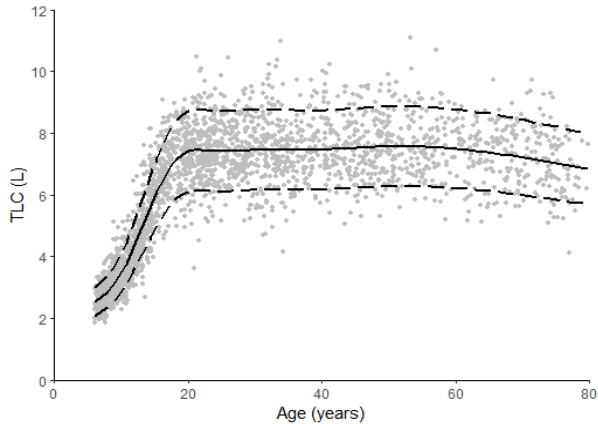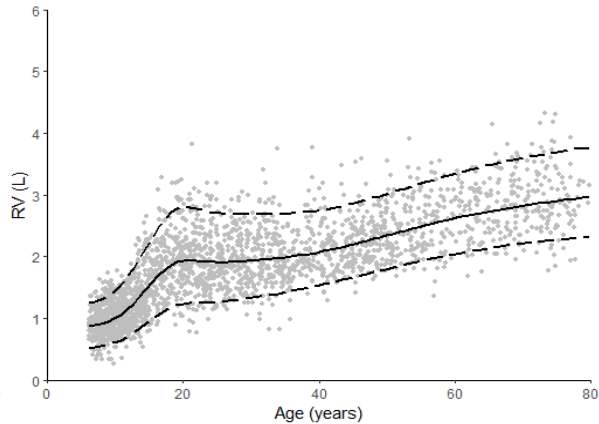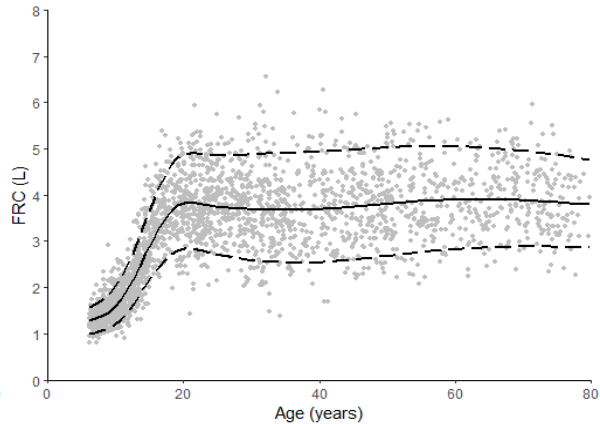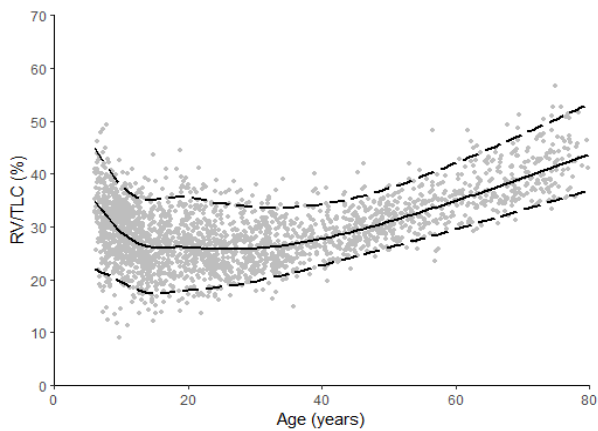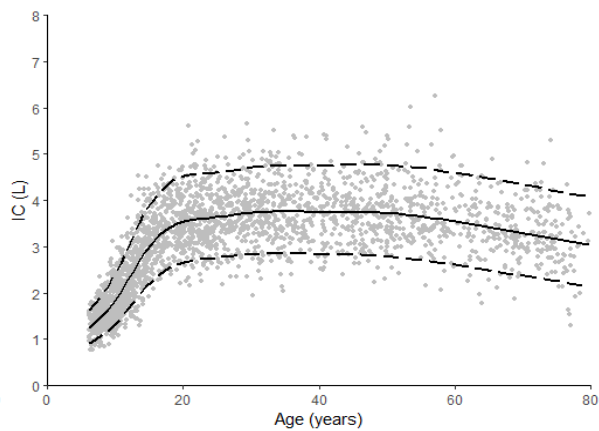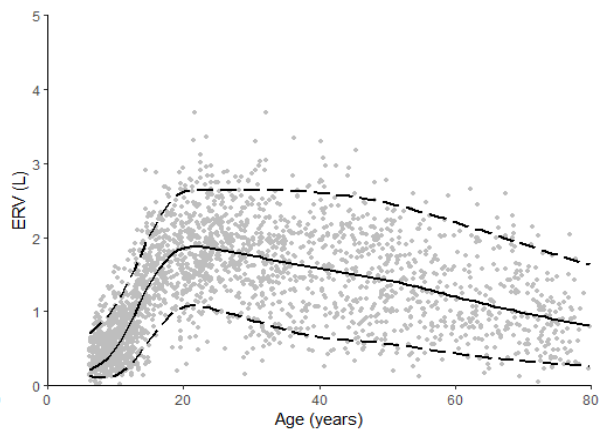

Supplement: Supplementary file 1 — Additional file 1. Mean predicted lung volumes males [file 12931_2024_2782_MOESM1_ESM.pdf]

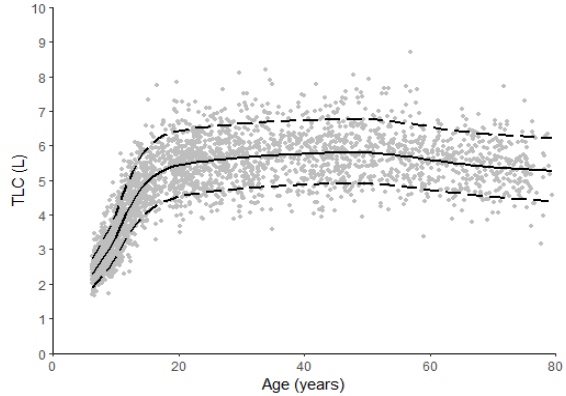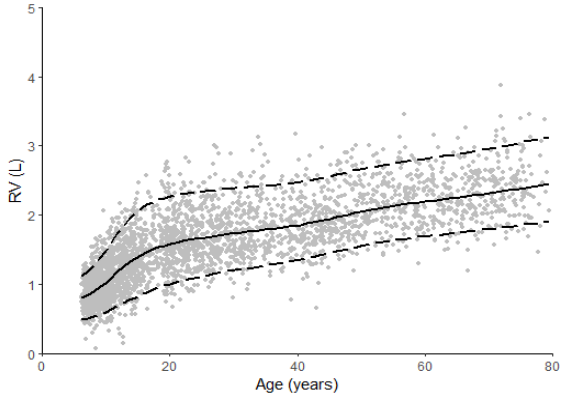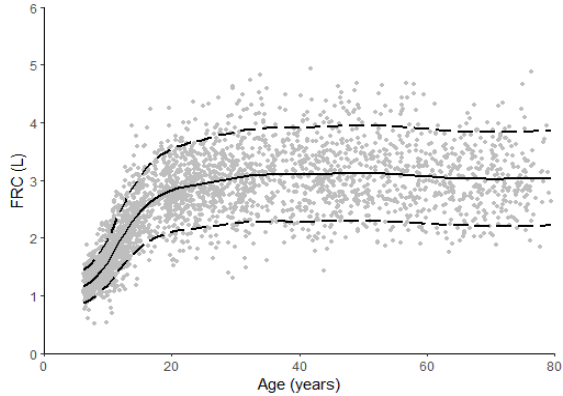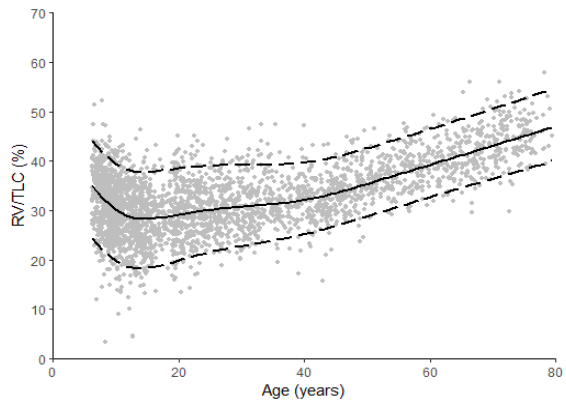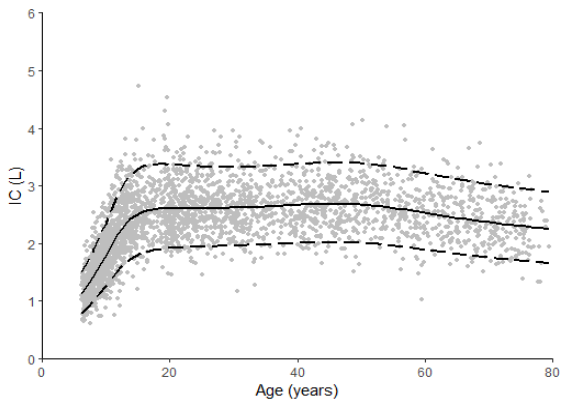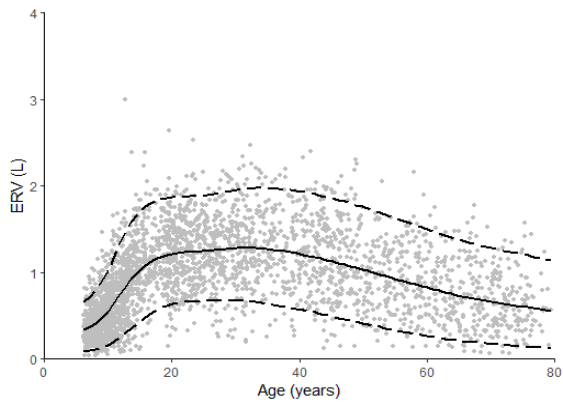

Supplement: Supplementary file 2 — Additional file 2. Mean predicted lung volumes females [file 12931_2024_2782_MOESM2_ESM.pdf]
